# Cognitive and Neuroimaging Profiles of Individuals With Dual Decline in Memory and Gait

**DOI:** 10.1093/geroni/igaa057.2768

**Published:** 2020-12-16

**Authors:** Qu Tian, Susan Resnick, Christos Davatzikos, Stephanie Studenski, Luigi Ferrucci

**Affiliations:** 1 National Institute on Aging, Bethesda, Maryland, United States; 2 University of Pennsylvania, Philadelphia, Pennsylvania, United States; 3 University of Pittsburgh, Pittsburgh, Pennsylvania, United States

## Abstract

Across 6 aging cohorts we showed that dual decline in memory and gait speed was associated with increased risk of dementia compared to memory or gait decline only. We now characterize dual decliners. Using longitudinal BLSA data, we examined associations of phenotypic groups with changes in cognition, depressive symptoms, and brain volumes in areas important for cognitive (dorsolateral prefrontal, medial temporal) and motor functions (precentral gyrus,striatum,thalamus,anterior cingulate cortex) using linear mixed effects models (usual agers=reference), adjusting for covariates. Compared to usual agers, dual decliners had faster decline in card rotation score, greater increase in CES-D, and greater atrophy in thalamus and anterior cingulate cortex. Rates of change in these parameters did not differ among the other three groups. Dual decliners experience faster decline in visuospatial ability, greater atrophy in selected motor areas, and greater increase in depressive symptoms, suggesting potential mechanisms underlying increased dementia risk in dual decliners.

